# Guanylyl Cyclase-G Modulates Jejunal Apoptosis and Inflammation in Mice with Intestinal Ischemia and Reperfusion

**DOI:** 10.1371/journal.pone.0101314

**Published:** 2014-07-03

**Authors:** Hui-Chen Lo, Ruey-Bing Yang, Chien-Hsing Lee

**Affiliations:** 1 Department of Nutritional Science, Fu Jen Catholic University, New Taipei City, Taiwan; 2 Institute of Biomedical Sciences, Academia Sinica, Taipei, Taiwan; 3 Division of Pediatric Surgery, Changhua Christian Hospital, Changhau, Taiwan; 4 Graduate Institute of Medical Sciences, Chang Jung Christian University, Tainan, Taiwan; National University of Singapore, Singapore

## Abstract

**Background:**

Membrane bound guanylyl cyclase-G (mGC-G), a novel form of GC mediates ischemia and reperfusion (IR)-induced renal injury. We investigated the roles of mGC-G in intestinal IR-induced jejunal damage, inflammation, and apoptosis.

**Materials and methods:**

Male C57BL/6 wild-type (WT) and mGC-G gene knockout (KO) mice were treated with a sham operation or 45 min of superior mesenteric arterial obstruction followed by 3, 6, 12, or 24 h of reperfusion.

**Results:**

Sham-operated KO mice had significantly lower plasma nitrate and nitrite (NOx) levels and jejunal villus height, crypt depth, and protein expression of phosphorylated-nuclear factor-kappa-B (NF-κB), phosphorylated-c-Jun N-terminal kinases (JNK) 2/3, phosphorylated-p38, and B-cell lymphoma-2 (Bcl-2). They had significantly greater jejunal interleukin-6 mRNA, cytochrome c protein, and apoptotic index compared with sham-operated WT mice. Intestinal IR significantly decreased plasma NOx in WT mice and increased plasma NOx in KO mice. The jejunal apoptotic index and caspase 3 activities were significantly increased, and nuclear phosphorylated-NF-κB and phosphorylated-p38 protein were significantly decreased in WT, but not KO mice with intestinal IR. After reperfusion, KO mice had an earlier decrease in jejunal cyclic GMP, and WT mice had an earlier increase in jejunal proliferation and a later increase in cytosol inhibitor of kappa-B-alpha. Intestinal IR induced greater increases in plasma and jejunal interleukin-6 protein in WT mice and a greater increase in jejunal interleukin-6 mRNA in KO mice.

**Conclusions:**

mGC-G is involved in the maintenance of jejunal integrity and intestinal IR-induced inflammation and apoptosis. These results suggest that targeting cGMP pathway might be a potential strategy to alleviate IR-induced jejunal damages.

## Introduction

Intestinal ischemia and reperfusion (IR) syndrome is a life-threatening dilemma caused by diverse events, including intestinal intussusception and transplantation, neonatal necrotizing enterocolitis, acute mesenteric arterial occlusion, and hemodynamic shock [1]. Intestinal ischemia results in impaired blood flow and local and systemic inflammation, but restoration of the blood flow intensifies ischemia-caused damage and leads to multiple organ failure (MOF) [2]. The pathophysiological changes in intestinal IR are closely associated with the changes in cytokines [3], nitric oxide (NO) [4], apoptotic pathways, and transcription factors [5].

Researchers demonstrated that intestinal IR-induced mucosal injury starts at 30 min and maximizes at 12 h of reperfusion [6], and the injury is associated with NO-cyclic guanosine monophosphate (cGMP) pathway [7]. The pro- and anti-apoptotic effects of cGMP, a secondary messenger generated by cytoplasmic soluble guanylyl cyclases (GC) and receptor-linked GCs, are mediated by the activation of c-Jun N-terminal kinases (JNK) and B-cell lymphoma-2 (Bcl-2) [8], [9]. Schulz et al. [10] found that a receptor-linked, membrane-bound GC-G (mGC-G) mRNA was predominantly expressed in the lung, kidneys, intestine, and skeletal muscle. This glycoprotein mGC-G exhibits marked cGMP-generating activity [11] and acts as an early signaling molecule in promoting apoptotic and inflammatory responses [12].

In intestinal IR, NO activates GCs to generate cGMP [13] and plays a dual role with cytotoxic and cytoprotective effects. For example, NO has been found to cause acute rejection in allotransplanted intestine [14]. Intestinal IR significantly increases tumor necrosis factor (TNF)-α, induces severe mucosal injury and cell apoptosis [5], and activates inflammatory response via nuclear factor-kappa-B (NF-κB) [15]. Intestinal IR-induced mucosal apoptosis is closely associated with increased cytochrome c secretion from mitochondria [16], [17] and activated caspase-9/caspase-3, JNK, and p38 mitogen-activated protein kinase (MAPKs) pathways [18]–[20]. The biological functions of mGC-G in the small intestine remain unclear.

In the present study, we hypothesized that the mGC-G gene plays critical roles in maintaining intestinal morphology and in modulating inflammatory response and intestinal damage. Therefore, the jejunal morphology was compared between mGC-G gene knockout (KO) and wild type (WT) mice. Plasma and jejunal inflammatory mediators, such as, nitrate and nitrite (NOx), interleukin-6, and NF-κB, and molecules of apoptotic pathways were evaluated in WT and KO mice with intestinal IR injury. These parameters were monitored at different time points within 24 h of reperfusion to determine the roles of mGC-G in regulating jejunal injury in intestinal IR.

## Materials and Methods

### Animals and experimental design

Animal facilities and protocols were approved by the Laboratory Animal Care and Use Committee of Changhau Christian Hospital, Changhua, Taiwan, with approval number CCH-AE-95010. Male C57BL/6J WT mice weighing 20 to 25 grams (8 weeks old) were supplied by the Laboratory Animal Center of the National Taiwan University, Taipei, Taiwan. Male C57BL/6J mice with mGC-G gene knockout, generated by Yang and colleagues [12], [21], were supplied by the Laboratory Animal Center of the Institute of Biomedical Sciences, Academia Sinica, Taipei, Taiwan. The genetic background of the WT and GC-G KO mice is comparable.

Mice were acclimated to animal facility for 1 week before surgery. After fasting overnight, mice were randomly assigned to 10 groups: WT mice with a sham-operation [WT-SH group] or 45 min of intestinal ischemia followed by 3, 6, 12, or 24 h of reperfusion [WT-IR3, WT-IR6, WT-IR12, or WT-IR24 groups, n = 9/group] and mGC-G gene KO mice with a sham-operation [KO-SH group] or with 45 min of intestinal ischemia followed by 3, 6, 12, or 24 h of reperfusion [KO-IR3, KO-IR6, KO-IR12, or KO-IR24 groups, n = 10/group].

### Intestinal ischemia and reperfusion procedure

Under general anesthesia, i.e., intramuscular injection with ketamine and xylazine (100 and 10 mg/kg body weight, respectively), the mice underwent a 2 cm midline laparotomy, and the superior mesenteric artery (SMA) was obstructed by micro-vascular clamp to induce intestinal ischemia [22]. During the course of the ischemia, the color of the small intestine changed from pink to dark red and brownish. After 45 min of ischemia, the micro-vascular clamp was removed from SMA to allow blood reperfusion and the return of bowel color. The abdominal incision of the mice was closed with a chromic gut suture for the muscle layer and with surgical staples for the skin. The mice were subsequently housed in an animal facility with free access to water and chow diet and executed after 3, 6, 12, or 24 h of blood reperfusion.

The survival rate of each group was 100%. At the execution time, the mice were anesthetized with intramuscular injections of ketamine and xylazine (150 and 15 mg/kg body weight, respectively). Blood was collected by cardiac puncture and serum and plasma were obtained. The entire small intestine was removed and placed on ice. The small intestine from the proximal 5 cm from the stomach to the distal 10 cm from the cecum was collected as the jejunum.

### Inflammatory mediators, cGMP, and caspase-3 activity

The plasma and jejunal levels of NOx, TNF-α, and interleukin-6 were measured with a commercial colorimetric (Cayman Chemical, Ann Arbor, MI) and enzyme-linked immunosorbent assay (ELISA) kits (R&D Systems, Inc., Minneapolis, MN). The cGMP levels and caspase-3 activity in the jejunum were measured using commercially colorimetric assay kits (Biomol Research Labs Inc., Plymouth Meeting, PA; R&D Systems, Inc., Minneapolis, MN).

### Jejunal mass and morphological changes

Two 3-cm pieces of the jejunum were dissected, rinsed thoroughly with cold saline, opened longitudinally to expose the intestinal epithelium, and scraped gently with glass slides to obtain the mucosal layer, as described by Noda et al [23]. The wet and dry weights of the jejunum and the amounts of protein (Pierce Chemical Co, Rockford, IL) and DNA [24] in the mucosa were analyzed.

One 1-cm piece of the jejunum was fixed in 10% buffered formalin for routine paraffin embedding, hematoxylin and eosin (H&E) staining, and morphometric measurements. The villus height, crypt depth, muscularis thickness, and villus density were determined as previously described [25]. At least 10 villus-crypt axes were measured per animal.

### Jejunal apoptotic and proliferation status

A Terminal deoxynucleotidyl transferase dUTP nick end labeling (TUNEL) kit, i.e., immunoflorescence staining, was used to identify the apoptotic cells (Hoffmann-La Roche Inc., Branford, CT) with counterstaining of 4′-6-Diamidino-2-phenylindole (DAPI) for nucleus in the jejunal sections. The apoptotic index was calculated as the number of TUNEL-positive cells per five villi.

For proliferation status, jejunal sections were stained with a mouse anti-rat Ki-67 antibody (SP6, Thermo Fisher Scientific Inc., Waltham, MA) at a dilution of 1∶100. For the negative control, the primary antibody was replaced by 0.5% bovine serum albumin in the immunohistochemistry procedures. The proliferation index was calculated as the percentage of Ki-67 positive nuclei per total number of nuclei in 10 crypts.

### Jejunal IL-6 mRNA expression

Total RNA (5 µg) prepared from the jejunum using the TRIzol reagent (Invitrogen, Carlsbad, CA) were used for each polymerase chain reaction (PCR). Quantitative real-time PCR (qRT-PCR) analysis was performed twice in duplicate (ABI PRISM 7700, Applied Biosystems Inc., Foster City, CA). Mouse glyceraldehyde-3-phosphate dehydrogenase (GAPDH) mRNA levels were used as controls. The primers used for the qRT-PCR were: mouse interleukin-6 forward, 5′-CTG CAA GAG ACT TCC ATC CAG TT-3′; mouse interleukin-6 reverse, 5′-GAA GTA GGG AAG GCC GTG G-3′; GAPDH forward, 5′-GGC AAA TTC AAC GGC ACAGT-3′; and GAPDH reverse, 5′-AAG ATG GTG ATG GGC TTC CC-3′. The relative expression ratio of the mGC-G transcript to the GAPDH transcript was calculated.

### Jejunal apoptotic protein expression

The cytosol and nuclear proteins were extracted from the jejunum using a commercial kit (NE-PER Nuclear and Cytoplasmic Extraction Kit, Thermo Fisher Scientific Inc., Rockford, IL). Jejunal protein (30 µg) was fractionated by 10% sodium dodecyl sulfate-polyacrylamide gel electrophoresis (SDS-PAGE) and transferred to hydrophobic polyvinylidene difluoride (PVDF) membrane. The primary mouse antibodies, including total and phosphorylated NF-κB, cytosol inhibitor of kappa-B-alpha (IκB-α), Bcl-2, Bax, cytochrome c, caspase-3, extracellular-signal-regulated kinases (ERK) 1/2, JNK1, JNK2/3, p38 and β-actin (Cell Signaling Technology, Inc., Danvers, MA; Santa Cruz Biotechnology Inc., Santa Cruz, CA) were used at dilutions of 1∶1000 to 1∶10,000. Horse radish peroxidase (HRP)-labeled anti-goat and anti-rabbit IgG antibodies (Santa Cruz Biotechnology Inc., Santa Cruz, CA) were used at dilutions of 1∶5000 to 1∶25,000. The signals were visualized by incubating in a chemiluminescent HRP substrate (ECL, Millipore Co., Bedford, MA) and exposure to X-ray film. The band densities were determined using a BioSpectrum CCD Imaging System (Ultra-Violet Products Ltd., Upland, CA) with normalization to the β-actin signal.

### Statistical analysis

The values were expressed as the means ± standard error of the mean (SEM). The comparisons of each parameter among the 5 WT groups and among the 5 KO groups were determined by one-way analysis of variance (ANOVA) using the SAS general linear model program. The group means were considered significantly different at P<0.05 as determined by protective least significant difference (LSD) technique when ANOVA indicates an overall significant time effect. To investigate the effects of the mCG-C gene on each parameter, KO mice were compared with WT mice at the same time point using Student’s t-test.

## Results

### The mGC-G gene plays important roles in maintaining jejunal morphology and modulating jejunal injury response

**Figure 1:**
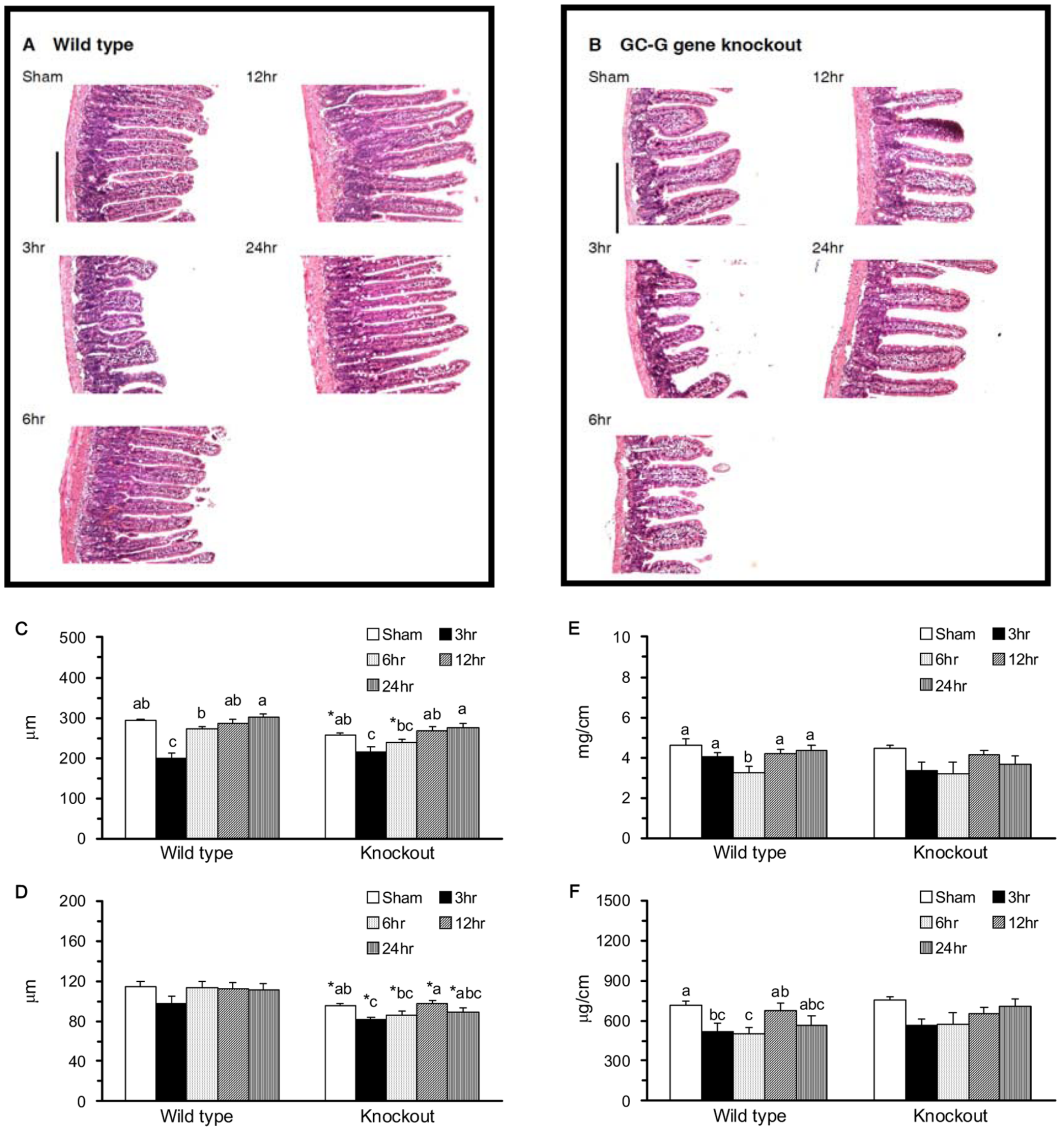
Jejunal morphology. H&E staining in WT (A) and KO (B) mice. Scale bars  = 25 µm. The average villus height (C), crypt depth (D), and mucosal dry weight (E) and protein content (F) per centimeter of jejunum. Means ± SEM, n = 10 for WT groups and n = 9 for KO groups. *P<.05, KO vs. WT mice with sham operation or with intestinal IR at the same time point (Student’s t-test,). Values with different superscripts indicate significant differences within all of the time points in WT and KO mice, individually (one-way ANOVA with LSD, P<.05).

The intestinal IR-induced jejunal atrophy was observed in the WT-IR3 (Figure 1A) and KO-IR3 groups (Figure 1B) and was reversed in the WT-IR6 and KO-IR12 groups. With sham operation, the KO-SH group had significantly lower jejunal villus density (P = 0.012), villus height (Figure 1C), and crypt depth (Figure 1D) than the WT-SH group. With intestinal ischemia, the WT-IR3 and KO-IR3 groups had significantly decreased villus heights and the IR-induced decrease in villus height was reversed in the WT-IR6 and KO-IR12 groups. The crypt depth was significantly decreased at 3 h of reperfusion and reversed at 12 h of reperfusion in KO, but not in WT mice. Neither the mGC-G gene nor intestinal IR had significant impact on muscularis thickness.

In the jejunal mass, the WT-IR6 and WT-IR3 groups had significantly decreased mucosal dry weight (Figure 1E) and protein content (Figure 1F), respectively, and these decreases were reversed in the WT-IR12 group. The KO-SH group had significantly decreased mucosal DNA content in the jejunum compared with the WT-SH group (P<.001). Intestinal IR did not have a significantly impact on mucosal DNA content in WT and KO mice.

### The mGC-G gene involves in the systemic and jejunal inflammatory responses

**Figure 2:**
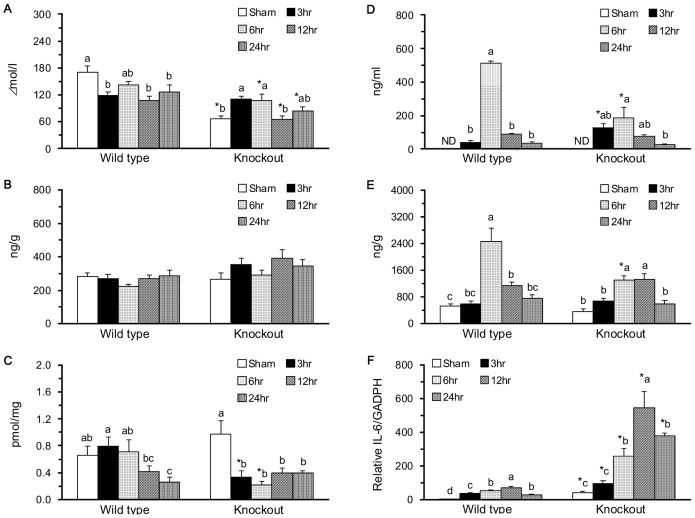
Nitrite/nitrate (NOx), cyclic GMP, and IL-6 protein and mRNA expression in the plasma and jejunum. Plasma NOx (A), jejunal NOx (B), jejunal cyclic GMP (C), plasma IL-6 (D), jejunal IL-6 protein (E), and the relative IL-6 mRNA expression in the jejunum after adjustment with GAPDH mRNA levels (F). Means ± SEM, n = 10 for the WT groups and n = 9 for the KO groups. *P<.05, KO vs. WT mice with sham operation or with intestinal IR at the same time point (Student’s t-test,). Values with different superscripts indicate significant differences within all of the time points in WT and KO mice, individually (one-way ANOVA with LSD, P<.05).

The KO-SH group had significantly lower plasma NOx concentrations compared with the WT-SH group (Figure 2A). With intestinal IR, the WT-IR3, WT-IR12, and WT-IR24 groups had significantly decreased plasma NOx compared with the WT-SH group. In contrast, the KO-IR3 and KO-IR6 groups had significantly increased plasma NOx compared with the KO-SH group. The jejunal contents of NOx (Figure 2B) and cyclic GMP (Figure 2C) were not significantly different between the WT-SH and KO-SH groups. Intestinal IR did not significantly affect the jejunal content of NOx in WT and KO mice. The jejunal content of cyclic GMP was significantly decreased in the WT-IR24 group compared with the WT-SH group (P<.001) and decreased in the KO-IR3, KO-IR6, KO-IR12, and KO-IR24 groups compared with the KO-SH group (P<.001).

The plasma IL-6 were undetectable in WT and KO mice and were significantly increased by intestinal IR (Figure 2D). The increase in plasma IL-6 was significantly lower in the KO-IR3 and KO-IR6 groups compared with the WT-IR3 and WT-IR6 groups, respectively. Plasma IL-6 was 1.7-fold greater in the WT-IR6 group than in the KO-IR6 group. The jejunal IL-6 contents were increased by 4-folds in the WT-IR6 and KO-IR6 groups compared with the WT-SH and KO-SH groups (Figure 2E). The IL-6 mRNA in the jejunum was more than 30-fold greater in the KO-SH group than in the WT-SH group (Figure 2F). Intestinal IR significantly increased the jejunal IL-6 mRNA in WT and KO mice and the increase was 7.7-fold greater in the KO-IR12 group than in the WT-IR12 group.

### The mGC-G gene plays a specific role in modulating the nuclear factor-κB (NF-κB) pathway in the jejunum in response to intestinal IR injury

**Figure 3:**
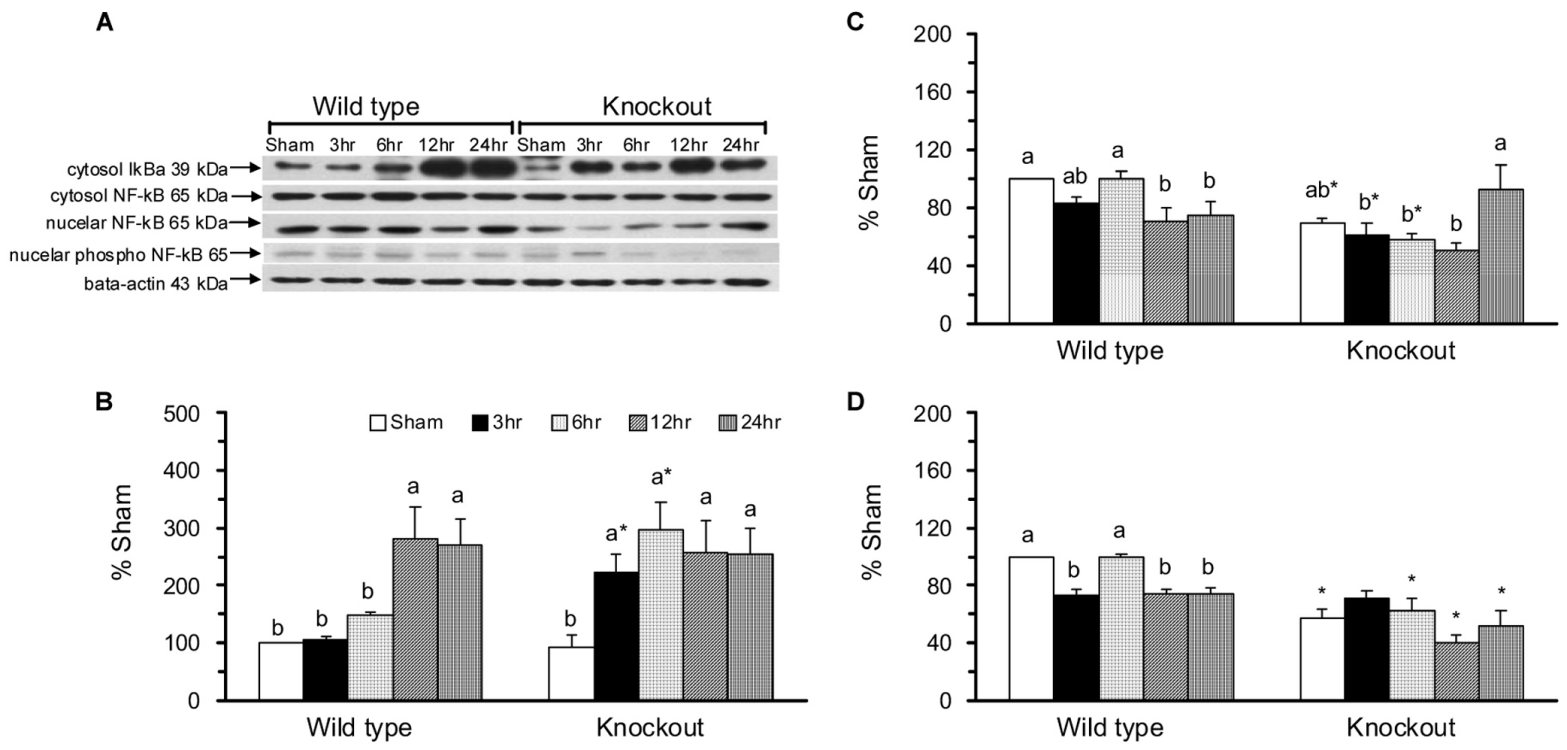
Molecules of the nuclear factor-kB (NF-κB) pathway in the jejunum. A western blot assay to detect cytosolic inhibitor of κB (IκB), nuclear total and phosphorylated NF-κB, and the internal control β-actin (A). Protein expression of cytosolic IκB (B), nuclear total (C), and nuclear active, phosphorylated (D) NF-κB. Protein quantification was carried out by densitometric analysis, normalized by the internal control β-actin, and calculated as the percentages of the WT-SH group. Means ± SEM, n = 10 for the WT groups and n = 9 for the KO groups. *P<.05, KO vs. WT mice with sham operation or with intestinal IR at the same time point (Student’s t-test,). Values with different superscripts indicate significant differences within all of the time points in WT and KO mice, individually (one-way ANOVA with LSD, P<.05).

The protein expression of cytosolic IκB and nuclear total and phosphorylated NF-κB are shown in Figure 3A. Cytosolic IκB was not significantly different between the WT-SH and KO-SH groups, while it was significantly increased in the WT-IR12 and WT-IR24 groups compared with the WT-SH group and increased in the KO-IR3, KO-IR6, KO-IR12, and KO-IR 24 groups compared with the KO-SH group (Figure 3B). The nuclear total and phosphorylated NF-κB were significantly lower in the KO-SH group than in the WT-SH group (Figure 3C and 3D). The total nuclear NF-κB was significantly decreased in the WT-IR12 and WT-IR24 groups and the phosphorylated nuclear NF-κB was significantly decreased in the WT-IR3, WT-IR12, and WT-IR24 groups compared with the WT-SH group. Intestinal IR did not significantly alter the expression of nuclear total and phosphorylated NF-κB in KO mice.

### The mGC-G gene is involved in jejunal apoptosis and proliferation in response to intestinal IR injury

**Figure 4:**
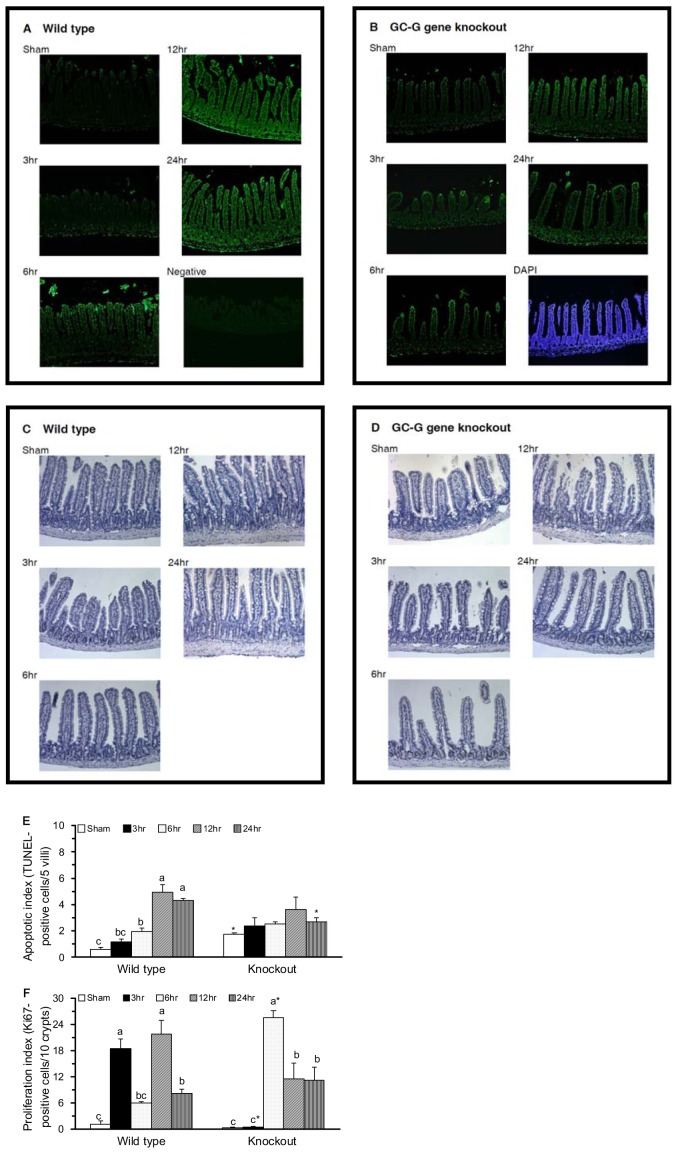
Immunofluorescence analysis of apoptotic cells and immunohistochemical analysis of cell proliferation in jejunal sections. *In situ* detection of apoptotic cells using TUNEL assay is shown with green fluorescein label in the jejunum of WT (A) and KO (B) mice. A negative control section treated with DNase without transferase is shown in the bottom right (A). The total nuclei stained with 4′,6-diamino-2-phenylindole (DAPI) were detected by UV light (blue) as shown in the bottom right (B). *In situ* detection of proliferative cells using Ki-67 staining is shown in the brown labeled jejunal crypts in WT (C) and KO (D) mice. The apoptotic index (E) and proliferation index (F) in per 5 villi and per 10 crypts, respectively. Means ± SEM, n = 10 for the WT groups and n = 9 for the KO groups. *P<.05, KO vs. WT mice with sham operation or with intestinal IR at the same time point (Student’s t-test,). Values with different superscripts indicate significant differences within all of the time points in WT and KO mice, individually (one-way ANOVA with LSD, P<.05).

The *in situ* immunofluorescent TUNEL analysis (Figure 4A and 4B) and immunohistochemical analysis of Ki-67 (Figure 4C and 4D) were used to determine the DNA damage and cell proliferation of jejunal enterocytes. The apoptotic index was significantly greater in the KO-SH group than in the WT-SH group and was significantly increased in the WT-IR6 group and further increased in the WT-IR12 and WT-IR24 groups compared with the WT-SH group (Figure 4E). There was no significant difference in the jejunal apoptotic index in KO mice with sham operations or intestinal IR.

The proliferation index, expressed as the Ki-67 positive cells, was not significantly different between the WT-SH and KO-SH groups (Figure 4F). In WT mice, the proliferation index was significantly increased during the reperfusion period and this increase was partially alleviated in the WT-IR6 and WT-IR24 groups. In KO mice, the proliferation index was significantly increased in the KO-IR6, KO-IR12, and KO-IR24 groups and this increase was partially alleviated in the KO-IR12 and KO-IR24 groups.

### The mGC-G gene modulates p38 activation in the jejunum in response to intestinal IR injury

**Figure 5:**
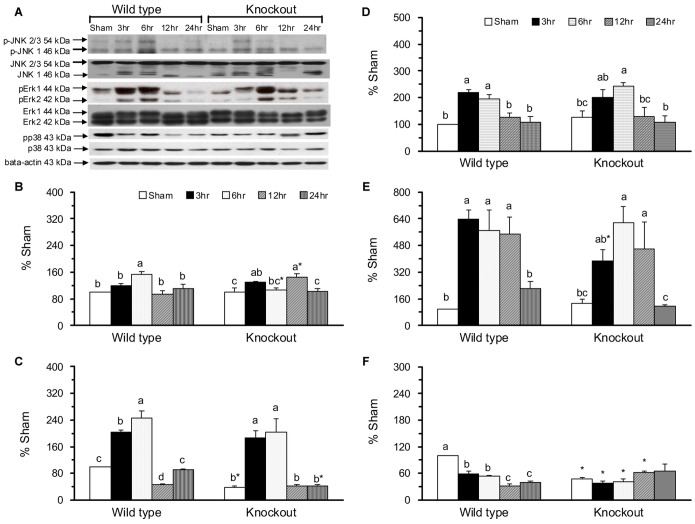
Molecules of the mitogen-activated protein kinases (MAPK) signaling pathway in the jejunum. A western blot assay to detect the protein expression of total and phosphorylated JNK, ERK, and p38 and the internal control β-actin (A). Protein expression of phosphorylated JNK1 (B), phosphorylated JNK 2/3 (C), phosphorylated ERK1 (D), phosphorylated ERK2 (E), and phosphorylated p38 (F). Protein quantification was carried out by densitometric analysis, normalized by the internal control β-actin, and calculated as the percentages of the WT-SH group. Means ± SEM, n = 10 for the WT groups and n = 9 for the KO groups. *P<.05, KO vs. WT mice with sham operation or with intestinal IR at the same time point (Student’s t-test,). Values with different superscripts indicate significant differences within all of the time points in WT and KO mice, individually (one-way ANOVA with LSD, P<.05).

The protein levels of MAPK signaling molecules were determined by a western blot assay (Figure 5A). The active, phosphorylated JNK1 was significantly increased in the WT-IR6 group compared with the WT-SH group and increased in the KO-IR3 and KO-IR12 groups compared with the KO-SH group (Figure 5B). The active, phosphorylated JNK2/3 was significantly decreased in the KO-SH group and increased in the WT-IR3 and WT-IR6 groups compared with the WT-SH group, and was increased in the KO-IR3 and KO-IR6 groups compared with the KO-SH group (Figure 5C).

The active, phosphorylated ERK1 was significantly increased in the WT-IR3 and WT-IR6 groups (Figure 5D), and phosphorylated ERK2 was significantly increased in the WT-IR3, WT-IR6, and WT-IR12 groups compared with the WT-SH group (Figure 5E). In KO mice, phosphorylated ERK1 was significantly increased in the KO-IR6 group, and phosphorylated ERK2 was significantly increased in the KO-IR6 and KO-IR12 groups compared with the KO-SH group.

The active, phosphorylated p38 was significantly decreased in the KO-SH group compared with the WT-SH group and was decreased in the WT-IR3, WT-IR6, WT-IR12, and WT-IR24 groups compared with the WT-SH group (Figure 5F). The intestinal IR-induced decrease in phosphorylated p38 was not found in KO mice.

### The mGC-G gene modulates intrinsic apoptotic and anti-apoptotic molecules in the jejunum in response to intestinal IR injury

**Figure 6:**
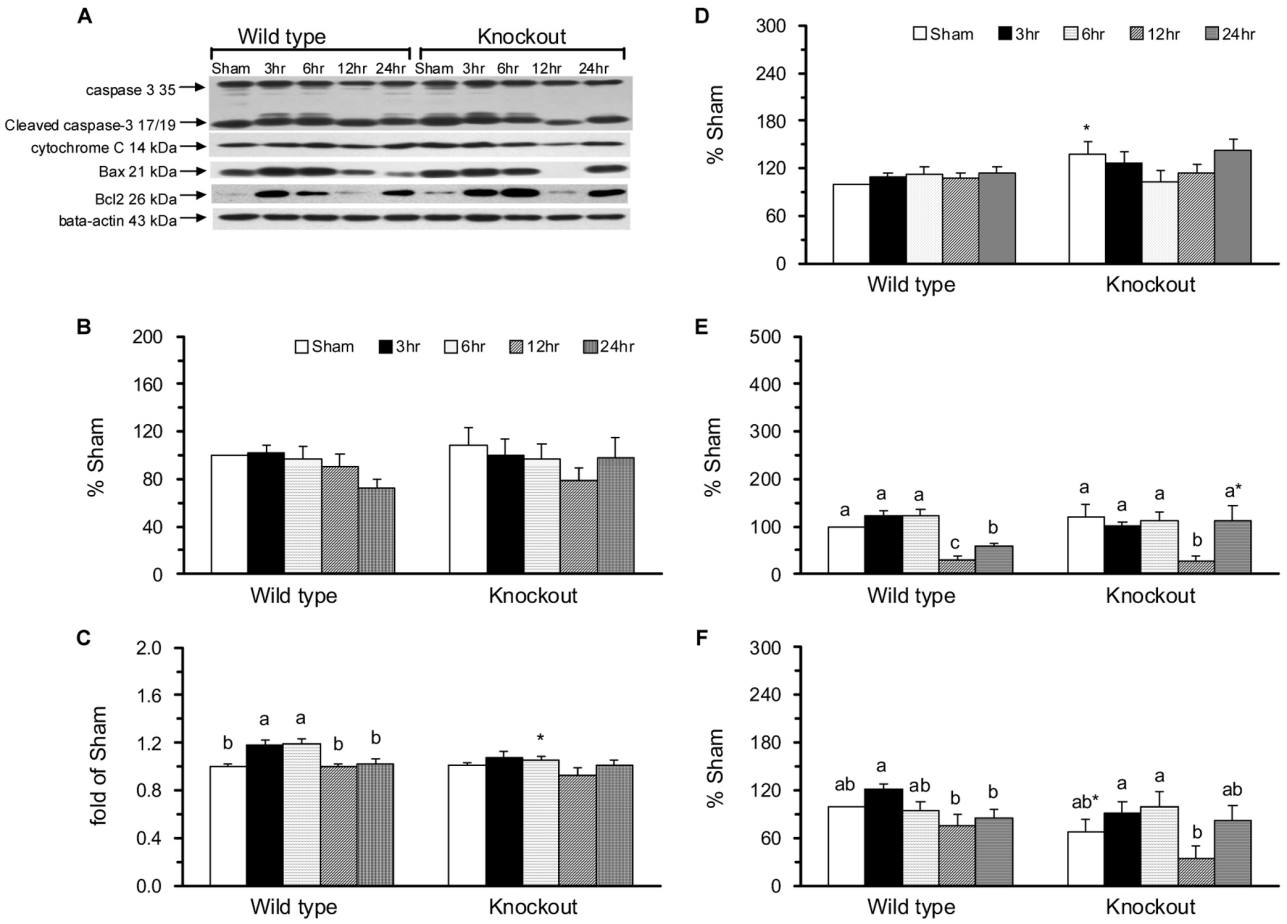
Molecules of the apoptotic intrinsic pathway in the jejunum. A western blot assay was employed to detect the protein expression of caspase 3 (Mw = 35 kDa), cleaved caspase 3, cytochrome c, Bax, Bcl-2, and the internal control β-actin (A). The protein expression of activated, cleaved caspase 3 (B), the activity of caspase 3 (C), and the protein expression of cytochrome c (D), apoptotic protein Bax (E), and anti-apoptotic protein Bcl-2 (F). Protein quantification was carried out by densitometric analysis, normalized by the internal control β-actin, and calculated as the percentages of the WT-SH group. Means ± SEM, n = 10 for the WT groups and n = 9 for the KO groups. *P<.05, KO vs. WT mice with sham operation or with intestinal IR at the same time point (Student’s t-test,). Values with different superscripts indicate significant differences within all of the time points in WT and KO mice, individually (one-way ANOVA with LSD, P<.05).

The molecules of the intrinsic apoptotic pathway, such as caspase 3, cleaved caspase 3, cytochrome c, Bax, and Bcl-2, were determined by western blot assay (Figure 6A). The inactive (35-kDa proenzyme) and active, cleaved caspase 3 (17/19 kDa, Figure 6B) were similar between the WT-SH and KO-SH groups and were not affected by intestinal IR in WT and KO mice. The activity of caspase 3 was significantly increased in the WT-IR3 and WT-IR6 groups compared with the WT-SH group (Figure 6C). Intestinal IR had no significant impact on the activity of caspase 3 in KO mice. The protein expression of cytochrome c, the upstream protein of caspase 3 in the apoptotic pathway, was significantly greater in the KO-SH group than in the WT-SH group and was not affected by intestinal IR in WT or KO mice (Figure 6D).

The intestinal IR-induced changes in the pro-apoptotic Bax were similar in WT and KO mice (Figure 6E). Bax was significantly decreased in the WT-IR12 and KO-IR12 groups compared with the WT-SH and KO-SH groups, respectively (Figure 6E), and the decrease was significantly reversed in the WT-IR24 and KO-IR24 groups. The protein expression of anti-apoptotic Bcl-2 was lower in the KO-SH group than in the WT-SH group and was not significantly altered by intestinal IR in WT or KO mice (Figure 6F).

## Discussion

Intestinal IR-induced mucosal injury and inflammation are the major causes of MOF and subsequent death. The glycoprotein mGC-G exhibits cGMP-generating activity and acts as an early apoptotic signaling molecule. When subjected to renal IR, the mGC-G gene KO mice had attenuated renal dysfunction, tubular cell apoptosis, and inflammatory response compared with WT mice [12]. Because GC-G mRNA is expressed in the small intestine [10], inhibition of cGMP might be a viable approach to protect from intestinal IR injury. Using mGC-G gene knockout mice either exposed to intestinal IR or not, we demonstrated that the mGC-G gene is involved in the maintenance of jejunal integrity and the modulation of intestinal IR-induced jejunal damage, apoptosis, and inflammation.

It has been demonstrated that cGMP is involved in the regulation of a broad range of physiological processes, such as muscle cell growth, contractility, and differentiation, pathophysiology of hypertension and vascular injury, gene expression at transcriptional and posttranscriptional levels, and pro- or anti-proliferative effects [9]. The receptor-linked mGC-G, an orphan receptor without a known ligand, may produce cGMP to perform physiological functions in tissues and organs. In the present study, KO mice had similar body weights and organ and tissue weights as WT mice, suggesting that mGC-G might not affect growth in mice. However, the sham-operated KO mice had significantly lower jejunal crypt depth, villus density and height (Figure 1), and mucosal DNA content compared with the sham-operated WT mice. These results suggest that the mCG-G gene plays specific roles in maintaining jejunal morphology and proliferation, which might be closely associated with the physiological function of cGMP.

There are 2 types of GCs, the cytoplasmic soluble GS catalyzed by NO and the membrane-bound, receptor-linked GC catalyzed by natriuretic peptides [26]. The soluble GS associated NO-cGMP pathway is important for the activation of NF-κB in inflammatory response [27]. The biphasic effect of NO on IL-6 production is closely associated with changes in the cGMP levels and NF-κB activity in human peripheral blood mononuclear cells.^23^ It is unclear if mGC-G may regulate inflammatory response via cGMP. In the present study, the sham-operated KO mice had lower plasma NOx, similar jejunal NOx, cGMP (Figure 2), and IL-6 (Figure 3) levels, and lower jejunal total and phosphorylated nuclear NF-κB and greater IL-6 mRNA. These results suggest that the GC-G gene might affect cytokine production at the translation level via a NO-cGMP-independent pathway and is involved in NF-κB pathway activation, which warrants further investigation.

The pro- and anti-apoptotic effects of cGMP have been described in different cell types [9]. A well-known receptor-linked GC, GC-C might regulate the intestinal barrier function via a cGMP-dependent signaling pathway [28]. In the GC-C gene KO mice with chemical-induced colitis, apoptosis, TNF-α, and IFN-g levels in intestinal epithelial cells were significantly reduced [28]. In the present study, the novel GC-G gene KO mice with sham operation had significantly increased apoptotic index (Figure 4E) and cytochrome c (Figure 6C) and decreased phosphorylated JNK2/3 (Figure 5C), p38 (Figure 5F), and nuclear NF-κB (Figure 3D) in the jejunum compared with WT mice with sham operation. These findings indicate that the GC-G gene is important in activating MAPK apoptotic molecules and NF-κB pathways and in alleviating intrinsic apoptosis in the jejunum under unstressed normal condition.

In the small intestine, IR may result in high production of NO, TNF-α, and IL-6 [5], [29], initiation of the NF-κB pathway [4], increases in the percentage of DNA fragmentation, and the activation of JNK and p38 MAPK [30], [31]. The intestinal IR-induced apoptosis and mucosal recovery are rapid processes that return back to baseline levels at 24 h of reperfusion [6]. In the present study, WT mice with intestinal IR had significantly increased plasma IL-6 protein, jejunal IL-6 protein and mRNA, caspase 3 activity, and protein expression of pro-apoptotic molecules, i.e., the active, phosphorylated JNK1 and JNK2/3, and anti-apoptotic molecules, i.e., the active, phosphorylated ERK1 and ERK2 (Figure 5). Though the plasma NOx and jejunal expressions of NF-κB (Figure 3), p38 (Figure 5), and Bax (Figure 6) were decreased, these mice had significantly decreased jejunal villus height and mucosa protein (Figure 1) and increased apoptotic index (Figure 4). These results confirm that intestinal IR may result in jejunal injury and apoptosis in WT mice.

The local release of NO is involved in the maintenance of intestinal permeability via activating soluble GC to produce cGMP for controlling vascular smooth muscle relaxation [13]. In the present study, the sham-operated KO mice had significantly lower plasma NOx and a similar amount of jejunal NOx and cGMP compared with the sham-operated WT mice. With intestinal IR, WT and KO mice had jejunal injury and inconsistent changes in plasma NOx and jejunal cGMP. The plasma NOx was decreased during the reperfusion period, and the jejunal cGMP content was decreased at 24 h of reperfusion in WT mice, while the plasma NOx was increased at 3 and 6 h of reperfusion, and the jejunal cGMP content was decreased during the reperfusion period in KO mice (Figure 2). KO mice had lower increases in plasma and jejunal IL-6 (Figure 2), earlier increases in cytosolic I-κB, and unaltered activation in NF-κB (Figure 3) compared with WT mice. These results suggest that the mGC-G gene might be crucial in the response to stress conditions, especially in activating the inflammatory response. Therefore, the mGC-G gene might be a clinical target for controlling the initiation of inflammatory responses in intestinal IR.

The protective effects of the soluble GC-cGMP pathway have been demonstrated in ischemia and reperfusion injury in the liver [32], heart [33], lung [34], and small intestine [7]. The inhibition of glycoprotein mGC-G production has been reported to have protective effects on renal injury, apoptosis, and the inflammatory response in mGC-G gene KO mice with renal IR [12]. In the present study, we found that knockout the mGC-G gene might alleviate the intestinal IR-induced jejunal apoptosis (Figure 4). This attenuated apoptotic response might be associated with inactivation of the p38 MAPK pathway (Figure 5) and caspase 3 activity (Figure 6) in KO mice. These results reveal that the GC-G gene is involved in the modulation of jejunal apoptosis via p38 MAPK and intrinsic apoptosis pathways in intestinal IR injury.

The present study has several limitations. Jejunal necrosis was not determined in this study. Although intestinal IR might result in both apoptosis and necrosis, apoptosis is the major mode of cell death during intestinal IR [35]. For this reason, we chose to focus on the effects of the mGC-G gene on apoptosis instead of on necrosis. The intestinal IR-induced intrinsic apoptotic pathway is closely related to mitochondrial function. In the present study, we did not measure the structural and functional changes of the enterocyte mitochondria, but we found that the KO mice with sham operations had significantly increased secretion of cytochrome c and decreased Bcl-2 expression in the jejunum, suggesting an altered mitochondrial function. It would be of interest to elucidate the role of the mGC-G gene on modulating the function of mitochondria. The intestinal IR-induced MOF was not evaluated in the present study. It has been found that mGC-G mRNA is expressed in the target organs including the lung and kidneys [10]. The lower degree of jejunal damage and apoptosis imply that the mGC-G gene KO mice might have a lower chance of developing MOF after experiencing intestinal IR. Studies are needed to evaluate the possibilities of the GC-G gene and protein inhibitors in preventing the development of ischemia and reperfusion injury.

**Figure 7:**
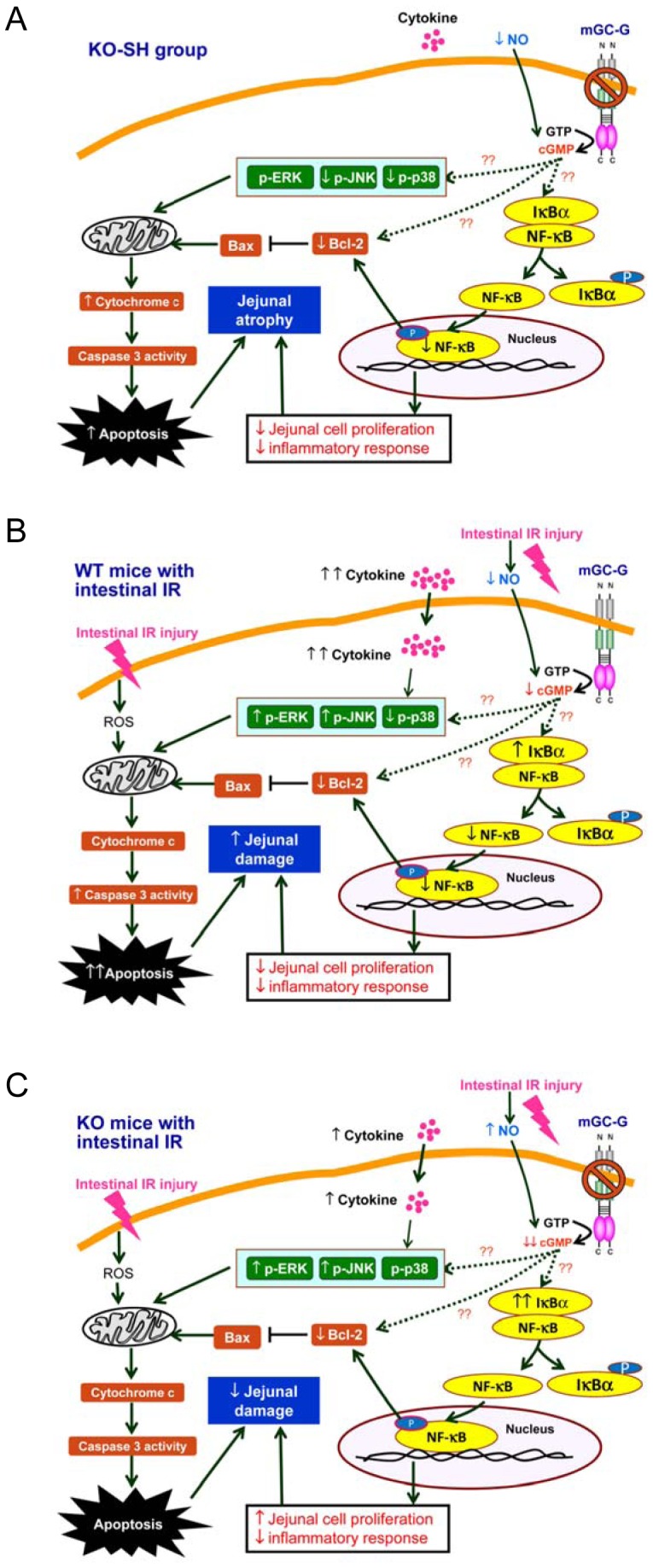
The hypothetical schemes of the relationships in GC-G/cGMP with major pathways contributed to jejunal atrophy in the KO-SH group (A) and to the intestinal IR-induced jejunal damage in WT (B) and KO (C) mice. The dash lines with symbols “??” represent unknown links between cGMP and the downstream proteins and pathways.

The mechanisms of mGC-G gene in regulating jejunal atrophy and eliminating jejunal damage in mice with intestinal IR are highly complex and sophisticated, involving GC-G/cGMP, NO, NF-κB, cytokines, caspases, and MAPK pathways. The results of the present study reveal the relationships of GC-G/cGMP with these pathways without knowing the exact links. The schematic cascades described the possible roles of mGC-G gene in maintaining jejunal morphology is shown in Figure 7A. In KO mice, mGC-G gene knockout may result in decreased circulating NO, inactivated NF-κB pathway, decreased cell proliferation, and activated mitochondrial intrinsic apoptotic pathway. These changes result in the deceased jejunal mass and structure. The hypothetical scheme of the major pathways involved in the intestinal IR-induced jejunal damage in WT and KO mice are shown in Figure 7B and 7C, respectively. When suffered with intestinal IR, the decreased NO and cGMP may inactivate NF-κB pathway and the increased IL-6 may activate MAPK pathway and mitochondrial intrinsic apoptotic pathway, which result in severe jejunal damage in WT mice (Figure 7B). In KO mice, intestinal IR increases NO may result in further decreases in cGMP and attenuated activation of MAPK pathway and mitochondrial intrinsic apoptotic pathway which result in mild jejunal damage (Figure 7C).

The results of the present study revealed that the mGC-G gene plays important roles in maintaining jejunal morphology, especially the villi and crypts, and modulating the jejunal injury response, as shown by the decrease in enterocyte numbers and proliferation and reduced sensitive to intestinal IR injury in KO mice compared with WT mice. The mGC-G gene might be involved in the systemic and jejunal inflammatory responses, as evidenced by the decreased protein production of inflammatory mediators, such as IL-6, and non-activation of the NF-κB pathway, though IL-6 mRNA expression was elevated in the mGC-G gene KO mice. The attenuated apoptosis and delayed proliferation of the jejunum in KO mice with intestinal IR suggest that the mGC-G gene acts as a modulator for the stress response in the jejunum. The increased cytochrome c and decreased Bcl-2 in KO mice with sham operations, and the delayed ERK, non-responsive p38 signaling, and unaltered caspase 3 activity in KO mice with intestinal IR injury reveal that the GC-G gene is important in modulating the balance of apoptosis and anti-apoptosis in the jejunum. In conclusion, mGC-G is involved in the maintenance of jejunal integrity and intestinal IR-induced apoptotic and inflammation. The pharmacologic modulation of cGMP production of its signal transduction might provide a viable strategy to alleviate intestinal ischemia and reperfusion-induced jejunal damages.
